# Cantharidal Collodion

**Published:** 1867-12

**Authors:** 


### CANTHARIDAL COLLODION.

WE have recently been using this preparation topically in the treatment of acute Dental periostitis, and with very decided success.

The method of application is very simple. After having dried the surface of the gum about the tooth or root affected, with a napkin or bibulous paper, the application may be made with a camel's hair brush, or perhaps just as well with a portion of cotton or lint upon an instrument; the lip or cheek should be held away, and the moisture prevented from passing over the surface, till the ether has evaporated and the artificial cuticle formed.

Usually within a few moments after the application, the pain ceases, counter irritation takes place at once, and within a few hours blistering occurs, when periostitis is effectually relieved. This preparation has given such prompt and entire relief in almost every instance, that we regard it as one of the very best agents employed in the treatment of this affection. Our experience certainly warrants us in suggesting to the profession to test it. We shall be glad to hear of the results of its use in the hands of others.

It may be obtained of druggists generally. T.

